# Free-water diffusion tensor imaging detects occult periependymal abnormality in the AQP4-IgG-seropositive neuromyelitis optica spectrum disorder

**DOI:** 10.1038/s41598-021-04490-3

**Published:** 2022-01-11

**Authors:** Minchul Kim, Kyu Sung Choi, Ryoo Chang Hyun, Inpyeong Hwang, Tae Jin Yun, Sung Min Kim, Ji-hoon Kim

**Affiliations:** 1 Department of Radiology, Kangbuk Samsung Hospital, Sungkyunkwan University School of Medicine, Seoul, Republic of Korea; 2 Department of Radiology, Seoul National University Hospital, Seoul National University College of Medicine, Seoul, Republic of Korea; 3 Department of Neurology, Seoul National University Hospital, Seoul National University College of Medicine, Seoul, Republic of Korea

**Keywords:** Neurology, Neurological disorders, Demyelinating diseases, Multiple sclerosis

## Abstract

To compare free-water corrected diffusion tensor imaging (DTI) measures in the normal-appearing periependymal area between AQP4-IgG-seropositive NMOSD and multiple sclerosis (MS) to investigate occult pathophysiology. This prospective study included 44 patients (mean age, 39.52 ± 11.90 years; 14 men) with AQP4-IgG-seropositive NMOSD (n = 20) and MS (n = 24) who underwent DTI between April 2014 and April 2020. Based on free-water corrected DTI measures obtained from normal-appearing periependymal voxels of (1) lateral ventricles and (2) the 3rd and 4th ventricles as dependent variables, MANCOVA was conducted to compare the two groups, using clinical variables as covariates. A significant difference was found between AQP4-IgG-seropositive NMOSD and MS in the 3rd and 4th periependymal voxels (λ = 0.462, *P* = 0.001). Fractional anisotropy, axial diffusivity was significantly decreased and radial diffusivity was increased in AQP4-IgG-seropositive NMOSD in post-hoc analysis, compared with MS (*F* = 27.616, *P* < 0.001, *F* = 7.336, *P* = 0.011, and *F* = 5.800, *P* = 0.022, respectively). Free-water corrected DTI measures differ in the periependymal area surrounding the diencephalon and brain stem/cerebellum between MS and NMOSD, which may suggest occult white matter injury in areas with distribution of AQP-4 in NMOSD.

## Introduction

Although discordant with traditional concepts, many studies have revealed that there is brain involvement in up to 60% of neuromyelitis optica spectrum disorder (NMOSD) patients^1^ and is recognizable as a brain MRI abnormality. Hypothalamic and brainstem lesions, such as area postrema lesions, have been known to be specific to NMOSD and somewhat atypical in multiple sclerosis (MS)^1^. Circumventricular involvement is not only confined to imaging findings but also related to clinical symptoms such as area postrema syndrome and the pathophysiology of NMOSD^2^.

Dramatically advancing our understanding of NMOSD, autoimmune antibody of aquaporin-4 (AQP-4), a water channel in foot processes of astrocytes that comprise the blood–brain barrier (BBB)^3^, has been revealed to be a key pathology of NMOSD, a “water channelopathy”, which was reflected in the revised criteria in 2015^4^. AQP-4 is largely distributed in periependymal surfaces of the lateral, third, and fourth ventricles, which has been confirmed in normal human brain tissue, as observed in other mammals, by immunolocalization studies^2,3^. Interestingly, lesions specific to NMOSD are more frequently localized in the periependymal areas surrounding the diencephalon and brainstem/cerebellum, including the hypothalamus, periaqueductal gray, and area postrema (i.e., the third and fourth ventricles) than around the lateral ventricles (i.e., lateral periventricular area), which corresponds to the AQP-4 channel distribution^3^. A total of 73–90% of NMOSD patients diagnosed based on the 2015 International Panel for NMO Diagnosis^4^ have aquaporin-4 antibodies (AQP4-IgG). Hereafter, we use the term “NMOSD group” to refer to NMOSD with AQP4-IgG^5,6^.

Recent updates of the clinical diagnostic criteria for both NMOSD and MS underscored the role of MRI for occult detection or diagnosis of the disease because many studies have revealed that early intervention for MS using drugs such as interferon might improve outcomes^7–9^. However, differential diagnosis of NMOSD and MS is crucial in that $\upbeta$-interferon, a disease-modifying treatment for relapsing–remitting multiple sclerosis (RRMS), may aggravate NMOSD^10,11^. Moreover, recurrent episodes/attacks in NMOSD resemble the clinical manifestation of RRMS (e.g., with optic neuritis and myelitis)^12^, which might confuse neurologists and lead to misdiagnosis.

Diffusion tensor imaging (DTI) is a specific type of modeling of diffusion weighted imaging (DWI) datasets^13^. The basic concept behind DTI is that it is possible to infer the tissue properties using eigenvalues and based on the water molecules diffusing differently along the structure of the tissues: 1) molecular diffusion rate–mean diffusivity (MD) or apparent diffusion coefficient (ADC); 2) directional preference of diffusion–fractional anisotropy (FA); 3) diffusion rate along the main axis of diffusion–axial diffusivity (AD); and 4) rate of diffusion in the transverse direction–radial diffusivity (RD)^13^. DTI is currently the best noninvasive MR technique to assess microstructural changes in white matter (WM) and has been used to investigate demyelinating and inflammatory diseases such as MS and NMOSD, even in the normal-appearing white matter (NAWM)^14–18^.

Based on the characteristic pathophysiology of and lesion distribution in NMOSD, we hypothesized that there might be occult damage in periependymal lining voxels, even before axonal damage becomes apparent in conventional sequences. We specified the NMOSD group as AQP4-IgG-seropositive to investigate the following question: “Can DTI detect abnormalities in the periependymal surface reflecting AQP4 status in the occult stage without definite T2 hyperintense changes in NMOSD with AQP4-IgG?”. To investigate our hypothesis, we analyzed periependymal lining voxels, which appear normal on T2-fluid attenuated inversion recovery (FLAIR) images, and used DTI to investigate the difference between MS and NMOSD to aid early, accurate differential diagnosis and enhance the deeper understanding of disease etiology. Further, it is important to correct for partial volume effect in the voxels surrounding ventricles because contamination of CSF has a large effect on DTI parameters. To overcome the effect of extracellular free-water on DTI metrics, we adopted a free-water correction algorithm that quantify and remove the contribution of extracellular free-water^19,20^.

## Materials and methods

### Patients

Consecutive patients who visited the Seoul National University Hospital (SNUH) MS-NMO clinic from April 2014 to April 2020 were prospectively enrolled in this study. This study was approved by the institutional review board of SNUH (IRB number: H-1310-083-528), and informed consent was obtained from each participant who was willing to enroll in this study. All processes related to this study were conducted in accordance with the Declaration of Helsinki. We conducted a single-centered prospective study, enrolling fifty-seven patients (*n* = 57) with the following inclusion/exclusion criteria: (1) diagnosed with MS according to the McDonald criteria; (2) diagnosed with NMOSD with AQP4-IgG according to the 2015 International Panel for NMO Diagnosis (IPND) criteria; (3) underwent MRI including diffusion tensor imaging (DTI) with three-dimensional isotropic T2-weighted FLAIR and three-dimensional magnetization-prepared rapid gradient-echo (3D MPRAGE) T1-weighted MRI, both of which allow thin-section and high-resolution imaging; and other patients were excluded if (1) the MR study was incomplete (n = 6); (2) there was an image processing failure (n = 1), and (3) clinical data were unavailable (n = 7). The IPND criteria were as follows: at least 1 of the core clinical characteristics, with no other better explanation for their symptoms (or exclusion of alternative diagnoses), and the six core clinical characteristics include (1) optic neuritis; (2) acute myelitis; (3) area postrema syndrome (i.e., nausea, vomiting, hiccups); (4) acute brainstem syndrome; (5) symptomatic narcolepsy or acute diencephalic syndrome with typical MRI lesion(s); and (6) symptomatic cerebral syndrome with typical MRI lesion(s)^4^. We did not exclude patients without definite MR lesions because we analyzed the normal-appearing periependymal region of the diencephalic and brain stem/cerebellar areas, not lesions with MR abnormalities. Finally, forty-four patients were enrolled in the present study (n = 24 in the MS group and n = 20 in the NMOSD group).

Clinical characteristics, including age, sex, expanded disability status scale (EDSS) scores, treatment history prior to MRI, and disease duration, were collected from the electronic medical record system of the hospital. Patients who did not receive immunomodulatory treatments such as steroid pulse therapy, and interferon, prior to MRI scan at our hospital, were defined as treatment-naïve patients. Propensity scores were matched by selecting the cases in the two groups, and the variables listed above were used as matching parameters using the ‘*matchit*’ R package (R core team, R foundation for statistical computing, Vienna, Austria)^21,22^. The matched cases included 20 patients for each MS and NMOSD group to ensure statistical significance.

### MRI techniques

All MR images were acquired using 3.0 T MR scanners (Ingenia CX, Philips Healthcare, Best, the Netherlands) with a conventional head gradient coil. T2 FLAIR imaging, T1-weighted imaging, and DTI were acquired with the following scan parameters: (1) three-dimensional (3D) isotropic fast-spin echo sagittal FLAIR T2-weighted sequence (repetition time [TR] = 4800 ms, echo time [TE] = 265 ms, inversion time = 1650 ms, echo train length = 175, field of view [FOV] = 230 mm, matrix = 230 × 230, and voxel size = 1 × 1 × 1 mm); (2) 3D high-resolution T1-weighted sequence (TR = 9.8 ms, TE = 4.5 ms, inversion time = 1650 ms, flip angle = 8°, FOV = 230 mm, matrix = 230 × 230, slice thickness = 0.5 mm, no gap, and voxel size = 1 × 1 × 0.5 mm) after administration of the intravenous gadolinium-based contrast agent, or gadoteridol (ProHance, Bracco Diagnostics, Inc., Princeton, NJ); and (3) spin-echo single-shot echo-planar imaging DWI sequence (TR = 9500 ms, TE = 75 ms, number of excitations = 1, matrix = 128 × 128, FOV = 230 × 230 mm, number of slices = 80, slice thickness = 2 mm, slice gap = 0 mm, orientation = axial, b = 1000 s/mm^2^ and one additional b0-volume). We used 32 nonlinear diffusion weighting gradient directions to estimate the intensity and direction of the diffusion anisotropy.

### MRI analysis

To isolate the normal-appearing voxels in the periependymal regions that are known to have high levels of aquaporin-4 expression, we used the following method.

**Figure 1:**
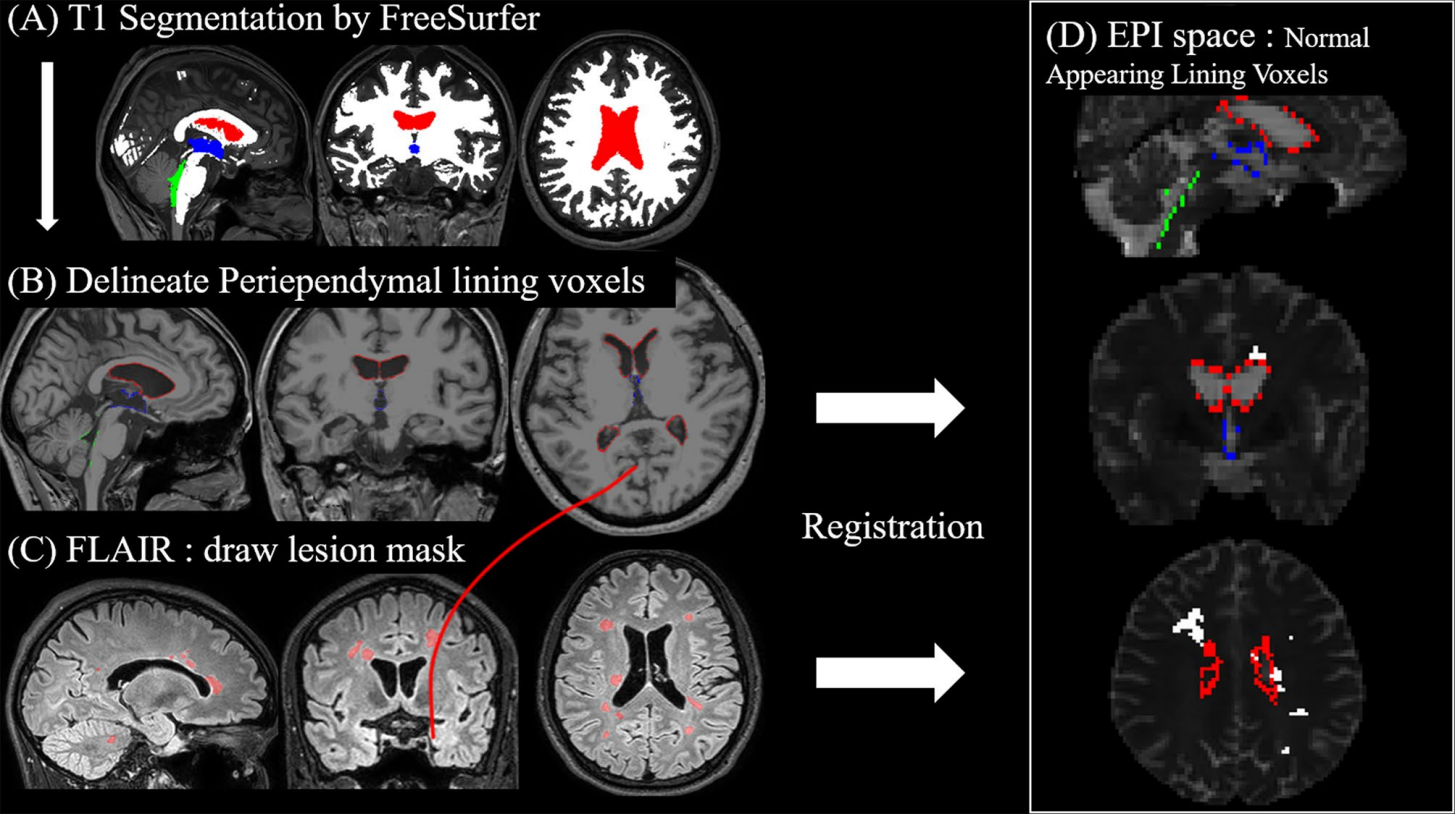
Schematic illustration of the analysis process. To delineate the diffusion tensor imaging (DTI) measures of the normal-appearing periependymal lining voxels, we analyzed the tissues as follows: (**A**) Using T1 imaging, we segmented the tissues into white matter, lateral ventricle (*red*), 3rd ventricle (*blue*), and 4th ventricle (*green*). (**B**) Then, we delineated the ventricle-lining voxels on the white matter mask using an open source MATLAB code^25^. (**C**) White matter lesion ROIs were manually drawn section by section on the T2-fluid attenuated inversion recovery (FLAIR) sequence. Then, the lesion masks were registered to T1 space, and both lesion mask and ventricular lining voxels in T1 space were registered to echo-planar imaging (EPI) space. (**D**) The figure shows the sagittal (*upper*), coronal (*middle*) and axial (*lower*) overlay of the periependymal lining voxels and the lesion masks on the nondiffusion-weighted image (B0). *Red*, lining voxels of the two lateral ventricles; *Blue*, lining voxels of the third ventricle; *Green*, lining voxels of the fourth ventricle; *White*, lesion masks.

Brain segmentation was performed in a completely automated manner via FreeSurfer (http://surfer.nmr.mgh.harvard.edu). Standard reconstruction procedures, which delineate gross brain anatomy into a series of cortical and subcortical labels, were used. Structures are labeled by using a complex algorithm combining information on image intensity, probabilistic atlas location, and the local spatial relationships between subcortical structures by the FreeSurfer “recon-all” function^23^. Brain regions numbered 46 (right cerebellar white matter), 7 (left cerebellar white matter) and ‘wm.mgz’ discovered by recon-all were added to create a white matter mask. Ventricular volumes were defined as the lateral (including the left and right lateral ventricles and choroid plexus), the third and fourth ventricles (Fig. 1A)^24^. To delineate the ventricular lining voxels, we used a method that can create a 1-voxel thick shell between two volumes implemented in MATLAB 2017b (The MathWorks, Inc., Natick, MA) (Fig. 1B)^25^. The white matter lesion region of interests (ROI) were manually drawn section by section on the FLAIR sequence by 2 authors (K.J.H., with 17 years of clinical experience in neuroradiology, and I.H., with 6 years of clinical experience in neuroradiology) (Fig. 1C).

We performed multiple registrations for each of the FLAIR-T1 and T1-diffusion image pairs by using the registration scheme provided by the ANTs software (antsRegistrationSyNQuick.sh^26,27^), which uses a mutual information metric. Then, the lesion ROIs in the FLAIR space and the ventricular lining shell in the T1 space were both transformed to the diffusion space. Since we wanted to compare the periependymal voxels that were not affected by the disease, we subtracted the lesion ROIs from the lining shell (Fig. 1D).

DWI data were preprocessed by denoising, followed by motion and eddy current correction using the MRtrix3 package^28^. For both visual and automated quality-assessment protocols for DWI, the temporal signal-to-noise ratio (tSNR) was assessed for each of the participants. With regard to tSNR, which is a method used to quickly screen the overall data quality, the participants’ lowest value was 7.57, which is above the suggested cutoff value (6.47) for poor data^26^. Free-water corrected DTI maps were calculated using an in-house MATLAB script (Fig. S1)^20,29^. Finally, we acquired the mean of the diffusion measures of each periependymal lining shell.

### AQP4 antibody assay

Because a cell-based assay is strongly recommended by the IPND criteria^4^, we confirmed the NMOSD group using a cell-based AQP4-Ab assay. Serum samples were tested by an in-house–developed M1-AQP4–transfected HEK-293 cell-based flow cytometry assay validated and certified by the College of American Pathology. Plasma protein obtained by the therapeutic plasmapheresis of a seropositive NMO patient (1:30 dilution, low positive) and the serum of a healthy control were used as positive and negative controls, respectively. The mean fluorescence intensity (MFI) values for FITC were measured as the binding of human IgG to the surface of live AQP4-expressing cells. The cutoff value for the fluorescence-activated cell sorting (FACS) assay and MFI index were determined by receiver operating characteristic curve analysis. To validate the accuracy of the in-house FACS assay for AQP4-IgG, serum samples of patients who agreed to undergo retesting were also tested for AQP4-Ab by conventional cell-based assay (O-CBA) at John Radcliffe Hospital (Oxford, UK)^30^ as an index test. For more detailed information, please refer to the previous study by Kim et al.^31^.

### Statistical analysis

Multivariate analysis of covariance (MANCOVA) was used to assess group differences between the MS and NMOSD groups using the mean FA, MD, AD and RD values as dependent variables. For assumption testing, we used the Mahalanobis distance to ensure multivariate normality and excluded one outlier in the NMOSD group^32,33^.

We used disease entity (MS/NMOSD) as the fixed factor, and age and EDSS scores were used as covariates since they significantly differed between groups. The multivariate effect of the independent variable was assessed by Wilks λ, which is a measure of the proportion of variance of the dependent variables that is accounted for by the independent variable. Adjusted means were calculated for each parameter on the basis of the linear models produced in the MANCOVA analysis.

Since MRI brain lesions that are characteristic of NMOSD are more commonly found around the third and fourth ventricles and the aqueduct of Sylvius than around the lateral ventricles^3^, we separately conducted two MANCOVAs: 1) diffusion tensor measures in the lateral ventricles as dependent variables and 2) diffusion tensor measures in the third and fourth ventricles as dependent variables.

Differences in continuous variables between groups were analyzed by 2-tailed unpaired t-tests, and differences in categorical variables between groups were analyzed by Fisher’s exact test. SPSS (Version 25.0, IBM, Armonk, NY) was used for all statistical analyses, and the results were considered significant at *P* < 0.05.

## Results

There were no significant differences between the clinical features (i.e. sex, EDSS scores, percentage of treatment-naïve patients, percentage of patients who have brainstem lesions, and disease duration) in the MS and NMOSD group, except for age (*P* = 0.001) (Table 1). Despite propensity matching, the NMOSD group showed significantly higher age and EDSS scores than the MS group, and we included those two variables as covariates in the following MANCOVA.

**Table 1:** Clinical characteristics of study population.

	NMOSD (n = 20)	MS (n = 24)	*P* value
Age (years)	46.16 ± 12.33	34.45 ± 7.94	0.001*
Gender (Male: Female)	2:17	8:12	.065
Disease duration (months)	46.2 ± 45.1	59.6 ± 71.5	.555
Treatment-naïve patients	8 (40.0%)	14 (58.3%)	.286
EDSS	3.31 ± 2.78	1.77 ± 1.43	.050*

EDSS, Expanded Disability Status Scale.

*P < 0.05 indicates statistical significance.

The MANCOVA analysis using the four diffusion tensor measures (FA, MD, AD, and RD) from third and fourth ventricle lining voxels as dependent variables, age and EDSS as covariates, and the disease entity as a fixed factor revealed a statistically significant difference between the patients with NMOSD and MS (λ = 0.462, *P* = 0.001) (Table 2). None of the covariates showed a significant overall effect on the extracted parameters (age: λ = 0.740, *P* = 0.142; sex: λ = 0.871, *P* = 0.621). Post hoc analyses revealed that the NMOSD group displayed significantly smaller fractional anisotropy, axial diffusivity and larger radial diffusivity (*F* = 27.616, *P* < 0.001, *F* = 7.336, *P* = 0.011, and *F* = 5.800, *P* = 0.022, respectively) in the voxels lining the third ventricle. Although increased mean diffusivity was reported in demyelinating disease^34^, there were no difference found in our result (Table 3). Descriptive statistics of the diffusion tensor measures of periependymal voxels for the third and fourth ventricles, respectively, are shown in Table 3.

**Table 2:** Results of group MANCOVA based on different ventricle locations.

Independent variables	Dependent variables	Covariates	Location	Wilk’s lambda	*df*	F	Partial Eta-squared	Observed power	*P* value
MS vs NMO	FA, MD, AD, RD	Age, EDSS	3rd and 4th ventricle	.462	6	5.619	.538	.988	< .001**
			Lateral ventricles	.790	6	2.835	.210	.625	.054

Dependent variables for each MANCOVA analysis are four diffusion tensor measures (i.e., FA, MD, AD, and RD) obtained in each specified location. *df* indicates degree of freedom for hypothesis testing. For statistical significance: ** P < .01.

**Table 3:** Descriptive statistics of the diffusion tensor metrics of periependymal voxels.

	NMOSD with AQP4-IgG	MS	*P*-value
**Third ventricle**			
Fractional anisotropy	.4962 ± 0.0390	.5389 ± 0.0363	< 0.001*
Mean diffusivity	.000632 ± 5.074E−05	.000641 ± 20628E−05	.705
Axial diffusivity	.000982 ± 5.926E−05	.001039 ± 10.212E−05	.011*
Radial diffusivity	.000458 ± 3.109E−05	.000442 ± 3.703E−05	.022*
**Fourth ventricle**			
Fractional anisotropy	.5533 ± 0.0591	.5972 ± 0.0515	.051
Mean diffusivity	.000598 ± 2.319E−05	.000623 ± 5.044E−05	.486
Axial diffusivity	.001027 ± 7.327E−05	.001118 ± 12.521E−05	.128
Radial diffusivity	.000384 ± 3.467E−05	.000375 ± 4.121E−05	.309

*P < 0.05 indicates statistical significance.

**Figure 2:**
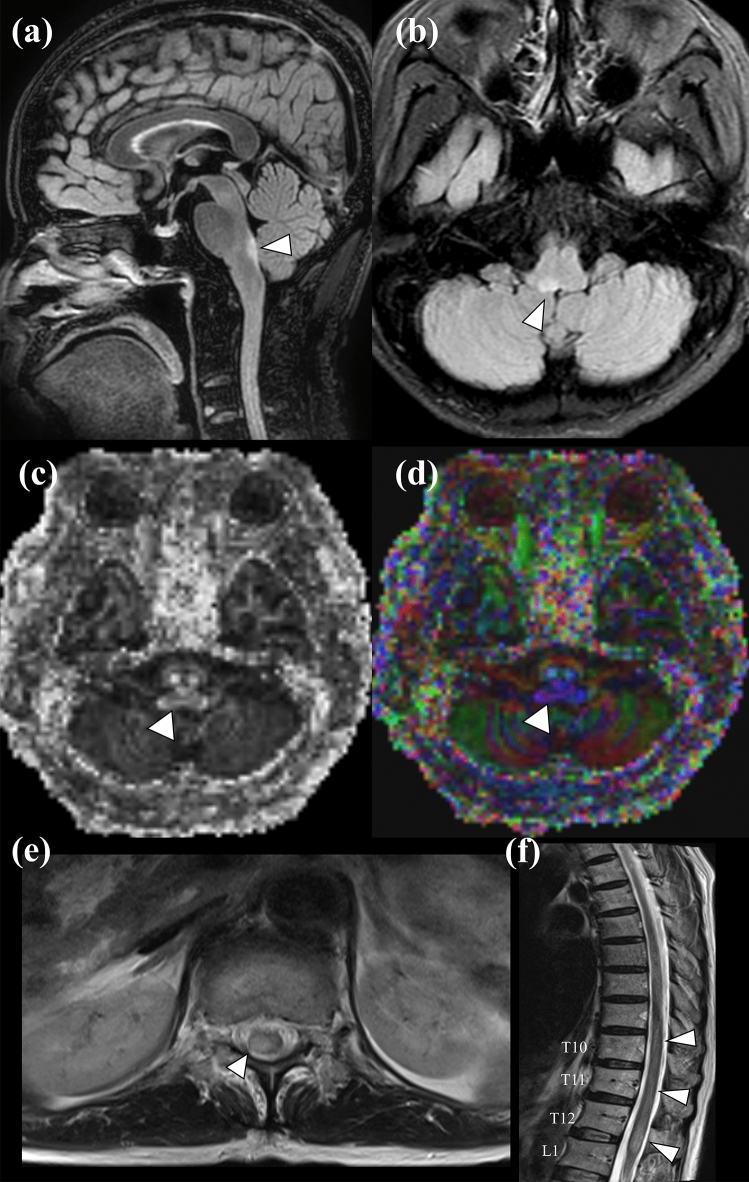
Representative patient with NMOSD with AQP4-IgG. A 57-year-old woman presented with intractable vomiting and gait disturbance (EDSS = 2; periependymal area of 4th ventricle: FA = 0.665, AD = 0.000609; periependymal area of 3rd ventricle: FA = 0.529, AD = 0.0011; periependymal area of left lateral ventricle: FA = 0.785, AD = 0.0008; and periependymal area of right lateral ventricle: FA = 0.780, AD = 0.00086). Note the periependymal T2 hyperintense lesion of right posterior medulla (or involving area postrema) on FLAIR image (*arrowhead*) (**a**) sagittal view, (**b**) axial view, as well as decreased (*dark area*) FA values on the (**c**) grayscale FA map, and heterogeneous colors on the (**d**) FA color-coded map at corresponding lesions (*arrowhead*). The patient also had an extensive, eccentric T2 hyperintense spinal cord lesion at the T10-L1 level (*arrowhead*) (**e**) axial view, (**f**) sagittal view.

**Figure 3:**
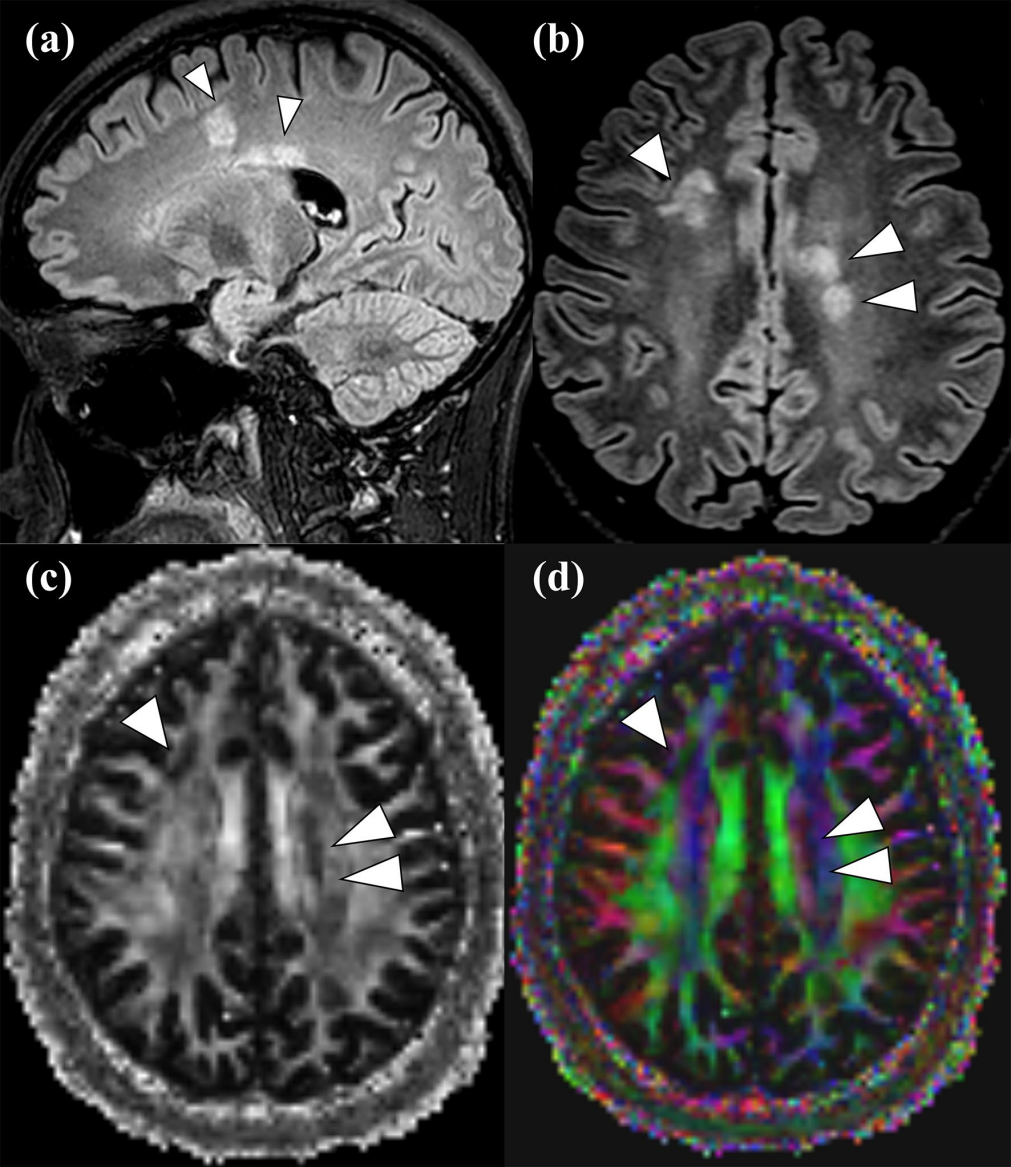
Representative patient with multiple sclerosis. A 23-year-old man presented with dysarthria and facial palsy (EDSS = 3; periependymal area of 4th ventricle: FA = 0.764, AD = 0.000643; periependymal area of 3rd ventricle: FA = 0.563, AD = 0.00134; periependymal area of left lateral ventricle: FA = 0.782, AD = 0.0004; and periependymal area of right lateral ventricle: FA = 0.765, AD = 0.00034). Note the multifocal periependymal T2 hyperintense lesions (*arrowhead*) on 3D FLAIR image (**a**) sagittal view, (**b**) axial view, as well as decreased (*dark area*) FA values on the (**c**) grayscale FA map, and heterogeneous colors on the (**d**) FA color-coded map at corresponding lesions (*arrowhead*).

The second MANCOVA using the four diffusion tensor measures (FA, MD, AD, and RD) from the lateral ventricle lining voxels did not reveal a significant difference between the patients with NMOSD and MS (λ = 0.790, *P* = 0.054). This indicated that white matter alterations in NMOSD differed from MS in periependymal regions of the diencephalon and brain stem/cerebellar area but not in the lateral periventricular regions. Representative examples from the NMOSD and MS groups are provided in Figs. 2 and 3, respectively.

While EDSS score is useful in MS, it may not be very useful in NMOSD. Because EDSS score focuses on ambulatory problem, NMOSD patient with sole area postrema syndrome (i.e. hiccups and vomiting) may even score almost 0 on EDSS. Therefore we additionally conducted MANCOVA without EDSS as covariate. The result was similar, revealing significant difference only from third and fourth ventricle lining voxels (λ = 0.632, P = 0.020, and for lateral ventricles, λ = 0.802, P = 0.298).

## Discussion

In the current study, we investigated occult changes in the periependymal area in patients with NMOSD with AQP4-IgG using free-water corrected DTI, an advanced MRI technique for detecting WM changes, compared to patients with MS, which is the most confusing differential diagnosis in clinical settings. Specifically, we analyzed normal-appearing voxels in the periependymal area to investigate whether diffusion tensor measures, including free-water corrected FA, MD, AD, and RD, differed between MS and NMOSD patients. In the MANCOVA analysis, there was a significant difference between MS and NMOSD patients on the combined four diffusion tensor measures (FA, MD, AD, and RD) from periependymal regions in the diencephalon (third ventricle) and brain stem/cerebellar areas including area postrema (fourth ventricle) (λ = 0.462, *P* = 0.001), whereas no significant difference was observed in lateral periventricular regions (λ = 0.790, *P* = 0.054), after controlling age and sex. Though not significant, there was trend in greater reduction of diffusion tensor measures in lateral periventricular regions in MS group than NMOSD group. In the post hoc analysis, FA and AD showed a significant decrease and RD increased in NMOSD patients compared with MS patients (Table 2, *F* = 27.616, *P* < 0.001, *F* = 7.336, *P* = 0.011, and *F* = 5.800, *P* = 0.022, respectively).

DTI variables are known to be related to alterations in structure pointing to specific injuries^13^. The FA is generally interpreted as a quantitative biomarker of white matter “integrity.”, and pathological studies tend to show a reduction of FA associated with neurodegenerative processes^35–37^. Also, the degree of anisotropy is often most strongly correlated with axon count and density^38^. Decrease in the axial diffusivity has been associated with axonal damage, and fragmentation in particular, whilst increase in radial diffusivity, which has been associated with axonal density, myelin integrity, axonal diameter, and fiber coherence, is correlated with myelin abnormalities^39,40^. Taken together, our results indicate that occult demyelination / axonal injury in periepndymal tissues was more prominent in the periependymal area surrounding the third and fourth ventricles than in lateral periventricular areas in NMOSD patients, and this difference can differentiate NMOSD from MS, reflecting the distribution of AQP-4, which is the key pathology associated with NMOSD.

In previous DTI studies, DTI measures obtained in NAWM^41,42^ and the optic radiation^43^ have revealed early alterations, representing widespread occult damage, even before apparent T2 hyperintense changes in NMOSD patients. In our study, we investigated whether occult alterations in diffusion tensor measures also take place in NAWM in the periependymal area, where AQP-4 is predominantly located. As a result, we discovered that alterations in DTI measures differed between NMOSD and MS patients only in periependymal tissues surrounding the third and fourth ventricles and not in lateral periventricular tissues. We suggest two possible explanations for this result. First, the predilection of AQP-4, a hallmark of NMOSD, for areas surrounding the third and fourth ventricles rather than lateral ventricles^3^ reflects different disease pathologies between NMOSD and MS. Another possible explanation is that both NMOSD and MS have occult damage in the NAWM of the lateral periventricular voxels because MS also involves the periventricular area, resulting in the typical appearance of ‘Dawson fingers’^2^.

Recent updates of the clinical diagnostic criteria for both NMOSD and MS underscore the role of MRI for early detection or diagnosis of the disease because many studies have revealed that early intervention for MS using drugs such as interferon might improve outcomes, whereas the same intervention can potentially aggravate NMOSD, which leads to the clinical importance of an early and accurate differential diagnosis of recurrent attacks of MS and NMOSD^10,11^. Our findings may lead to additional helpful information in the differential diagnosis of NMOSD and MS.

In a previous study by Pasquier et al.^44^, 7 T MRI failed to detect occult brain damage in NMOSD. They also analyzed lesion-free periependymal regions in NMOSD compared with MS. A recent study demonstrated that circulating autoantibodies against AQP4 can induce NMOSD in a rat model^45^. In immunohistochemical studies, periependymal (only third and fourth ventricles, not lateral ventricles) and hypothalamic localization of highly concentrated AQP4 was demonstrated in rodent and human brain, which also corresponds to frequently involved MRI brain lesions more commonly seen in NMOSD, which is different from MS^3^. Moreover, DTI has previously demonstrated occult damage in NAWM and the optic radiation, which lacks apparent hyperintense signal changes on T2-weighted images. Taken together, we can expect that there should be occult axonal injury without apparent demyelination, which can be detected as T2 hyperintense change, in predilected periependymal areas. We observed that DTI detected quantitative parametric alterations in specific periependymal areas as well as NAWM and the optic radiation in previous studies^41–43^, which revealed the capability of DTI to detect occult WM damage. Moreover, we specified the cohort as NMOSD with AQP4-IgG, and did not include those with NMOSD without AQP4-IgG, which was more consistent with our study goal. We not only matched the two groups using propensity score matching but also controlled for clinical characteristics such as age, sex, and EDSS scores as covariates using a multivariable approach. We also had a rather moderate sample size for a prospective, single-centered NMOSD and MS study (*n* = 20 and *n* = 24, respectively).

This study has a few limitations. First, the study lacked normal healthy controls and was a prospective but single-center study. Because the purpose of this study was to analyze the NMOSD-specific occult changes in periependymal areas, a normal healthy control group should be included in the cohort for comparison with the NMOSD group. However, the sample size meets the recommended size to reveal the statistical significance of between-group differences using MANCOVA^33^. In addition, relatively small sample size would also be a limitation of this study. We conducted power analysis using G*Power software with default effect size of pillai V = 0.4^46^, and got 32 total sample size required to get power > 80%. Although we met the power needed, however, future investigations will require a larger sample size, which can also lead to the development of a deep learning-based model for the differential diagnosis of MS and NMOSD. We used a fully automated segmentation and delineation method, which may be helpful in future studies regarding the replication of our findings.

In conclusion, the use of free-water corrected DTI allows periependymal lining voxels in the third and fourth ventricles to be used to differentiate MS from NMOSD. DTI measures differed between those two entities, suggesting occult white matter injury.

## Supplementary Information

Supplementary Information.

## Abbreviations

DTI: Diffusion tensor imaging
AQP4: Aquaporin 4
NMOSD: Neuromyelitis optica spectrum disorder
MS: Multiple sclerosis
MANCOVA: Multivariate analysis of covariance
FA: Fractional anisotropy
MD: Mean diffusivity
AD: Axial diffusivity
RD: Radial diffusivity
EDSS: Expanded disability status scale

## References

[1] Pittock SJ, Lennon VA, Krecke K, Wingerchuk DM, Lucchinetti CF, Weinshenker BG (2006). Brain abnormalities in neuromyelitis optica. Arch. Neurol..

[2] Dutra BG, da Rocha AJ, Nunes RH, Maia ACM (2018). Neuromyelitis optica spectrum disorders: Spectrum of MR imaging findings and their differential diagnosis. Radiographics.

[3] Pittock SJ, Weinshenker BG, Lucchinetti CF, Wingerchuk DM, Corboy JR, Lennon VA (2006). Neuromyelitis optica brain lesions localized at sites of high aquaporin 4 expression. Arch. Neurol..

[4] Wingerchuk DM, Banwell B, Bennett JL, Cabre P, Carroll W, Chitnis T (2015). International consensus diagnostic criteria for neuromyelitis optica spectrum disorders. Neurology.

[5] Hamid SH, Elsone L, Mutch K, Solomon T, Jacob A (2017). The impact of 2015 neuromyelitis optica spectrum disorders criteria on diagnostic rates. Mult. Scler..

[6] Hyun J-W, Jeong IH, Joung A, Kim S-H, Kim HJ (2016). Evaluation of the 2015 diagnostic criteria for neuromyelitis optica spectrum disorder. Neurology.

[7] Jang J, Nam Y, Choi Y, Shin N-Y, An JY, Ahn K-J (2020). Paramagnetic rims in multiple sclerosis and neuromyelitis optica spectrum disorder: A quantitative susceptibility mapping study with 3-T MRI. J. Clin. Neurol..

[8] Kim W, Park MS, Lee SH, Kim S-H, Jung IJ, Takahashi T (2010). Characteristic brain magnetic resonance imaging abnormalities in central nervous system aquaporin-4 autoimmunity. Mult. Scler. J..

[9] Kim W, Kim S-H, Kim HJ (2011). New insights into neuromyelitis optica. J. Clin. Neurol..

[10] Palace J, Leite MI, Nairne A, Vincent A (2010). Interferon Beta treatment in neuromyelitis optica: Increase in relapses and aquaporin 4 antibody titers. Arch. Neurol..

[11] Kim S-H, Kim W, Li XF, Jung I-J, Kim HJ (2012). Does interferon beta treatment exacerbate neuromyelitis optica spectrum disorder?. Mult. Scler. J..

[12] Matthews L, Marasco R, Jenkinson M, Küker W, Luppe S, Leite MI (2013). Distinction of seropositive NMO spectrum disorder and MS brain lesion distribution. Neurology.

[13] Soares J, Marques P, Alves V, Sousa N (2013). A hitchhiker's guide to diffusion tensor imaging. Front. Neurosci..

[14] Tackley G, Kuker W, Palace J (2014). Magnetic resonance imaging in neuromyelitis optica. Mult. Scler. J..

[15] Klawiter EC, Xu J, Naismith RT, Benzinger TL, Shimony JS, Lancia S (2012). Increased radial diffusivity in spinal cord lesions in neuromyelitis optica compared with multiple sclerosis. Mult. Scler. J..

[16] Rovaris M, Gass A, Bammer R, Hickman S, Ciccarelli O, Miller D (2005). Diffusion MRI in multiple sclerosis. Neurology.

[17] Rovaris M, Agosta F, Pagani E, Filippi M (2009). Diffusion tensor MR imaging. Neuroimaging Clin. N. Am..

[18] Rocca M, Cercignani M, Iannucci G, Comi G, Filippi M (2000). Weekly diffusion-weighted imaging of normal-appearing white matter in MS. Neurology.

[19] Pasternak O, Sochen N, Gur Y, Intrator N, Assaf Y (2009). Free water elimination and mapping from diffusion MRI. Magn. Reson. Med..

[20] Bergamino M, Walsh RR, Stokes AM (2021). Free-water diffusion tensor imaging improves the accuracy and sensitivity of white matter analysis in Alzheimer’s disease. Sci. Rep..

[21] Stuart EA, King G, Imai K, Ho D (2011). MatchIt: Nonparametric preprocessing for parametric causal inference. J. Stat. Softw..

[22] Team RC. R: A language and environment for statistical computing. Vienna, Austria; 2013.

[23] Fischl B, Salat DH, Busa E, Albert M, Dieterich M, Haselgrove C (2002). Whole brain segmentation: Automated labeling of neuroanatomical structures in the human brain. Neuron.

[24] Roberts D, Zhu X, Tabesh A, Duffy E, Ramsey D, Brown T (2015). Structural brain changes following long-term 6 head-down tilt bed rest as an analog for spaceflight. Am. J. Neuroradiol..

[25] Bonilha L, Gleichgerrcht E, Nesland T, Rorden C, Fridriksson J (2015). Gray matter axonal connectivity maps. Front. Psych..

[26] Avants BB, Tustison NJ, Song G, Cook PA, Klein A, Gee JC (2011). A reproducible evaluation of ANTs similarity metric performance in brain image registration. Neuroimage.

[27] Tustison NJ (2013). Explicit B-spline regularization in diffeomorphic image registration. Front. Neuroinform..

[28] Tournier J-D, Smith R, Raffelt D, Tabbara R, Dhollander T, Pietsch M (2019). MRtrix3: A fast, flexible and open software framework for medical image processing and visualisation. Neuroimage.

[29] Bergamino M, Kuplicki R, Victor TA, Cha YH, Paulus MP (2017). Comparison of two different analysis approaches for DTI free-water corrected and uncorrected maps in the study of white matter microstructural integrity in individuals with depression. Hum. Brain Mapp..

[30] Waters P, Jarius S, Littleton E, Leite MI, Jacob S, Gray B (2008). Aquaporin-4 antibodies in neuromyelitis optica and longitudinally extensive transverse myelitis. Arch. Neurol..

[31] Kim S-M, Waters P, Woodhall M, Kim Y-J, Kim J-A, Cheon SY (2017). Gender effect on neuromyelitis optica spectrum disorder with aquaporin4-immunoglobulin G. Mult. Scler. J..

[32] Tabachnick BG, Fidell LS, Ullman JB (2007). Using Multivariate Statistics.

[33] Pallant J (2013). SPSS Survival Manual.

[34] Rueda-Lopes FC, Hygino da Cruz LC, Doring TM, Gasparetto EL (2014). Diffusion-weighted imaging and demyelinating diseases: New aspects of an old advanced sequence. Am. J. Roentgenol..

[35] Assaf Y, Pasternak O (2008). Diffusion tensor imaging (DTI)-based white matter mapping in brain research: A review. J. Mol. Neurosci..

[36] Budde MD, Kim JH, Liang HF, Schmidt RE, Russell JH, Cross AH (2007). Toward accurate diagnosis of white matter pathology using diffusion tensor imaging. Magn. Reson. Med..

[37] Van Hecke W, Emsell L, Sunaert S (2015). Diffusion Tensor Imaging: A Practical Handbook.

[38] Beaulieu C, Jones DK (2011). What makes diffusion anisotropic in the nervous system. Diffusion MRI: Theory, Methods, and Applications.

[39] Concha L (2014). A macroscopic view of microstructure: Using diffusion-weighted images to infer damage, repair, and plasticity of white matter. Neuroscience.

[40] Song S-K, Sun S-W, Ramsbottom MJ, Chang C, Russell J, Cross AH (2002). Dysmyelination revealed through MRI as increased radial (but unchanged axial) diffusion of water. Neuroimage.

[41] Liu Y, Duan Y, He Y, Wang J, Xia M, Yu C (2012). Altered topological organization of white matter structural networks in patients with neuromyelitis optica. PLoS ONE.

[42] Kim SH, Kwak K, Hyun JW, Joung A, Lee S, Choi YH (2017). Diffusion tensor imaging of normal-appearing white matter in patients with neuromyelitis optica spectrum disorder and multiple sclerosis. Eur. J. Neurol..

[43] Zhao D-D, Zhou H-Y, Wu Q-Z, Liu J, Chen X-Y, He D (2012). Diffusion tensor imaging characterization of occult brain damage in relapsing neuromyelitis optica using 3.0 T magnetic resonance imaging techniques. Neuroimage.

[44] Pasquier B, Borisow N, Rasche L, Bellmann-Strobl J, Ruprecht K, Niendorf T (2019). Quantitative 7T MRI does not detect occult brain damage in neuromyelitis optica. Neurol. Neuroimmunol. Neuroinflamm..

[45] Hillebrand S, Schanda K, Nigritinou M, Tsymala I, Böhm D, Peschl P (2019). Circulating AQP4-specific auto-antibodies alone can induce neuromyelitis optica spectrum disorder in the rat. Acta Neuropathol..

[46] Faul F, Erdfelder E, Lang A-G, Buchner A (2007). G* Power 3: A flexible statistical power analysis program for the social, behavioral, and biomedical sciences. Behav. Res. Methods.

