# Supplementary material for: Free-water diffusion tensor imaging detects occult periependymal abnormality in the AQP4-IgG-seropositive neuromyelitis optica spectrum disorder
Source: Sci Rep. 2022 Jan 11;12:512. doi: 10.1038/s41598-021-04490-3 (PMC8752776; doi:10.1038/s41598-021-04490-3)
Supplement: Supplementary file 1 — Supplementary Information. [file 41598_2021_4490_MOESM1_ESM.docx]

**Free-water diffusion tensor imaging detects occult periependymal abnormality in the AQP4-IgG-seropositive neuromyelitis optica spectrum disorder**

Minchul Kim, M.D., Ph.D.^1*^, Kyu Sung Choi, M.D., Ph.D.^2*^, Ryoo Chang Hyun, M.D.^2^, Inpyeong Hwang, M.D.^2^, Tae Jin Yun, M.D.^2^, Sung Min Kim, M.D., Ph.D.^3†^, Ji-hoon Kim, M.D., Ph.D.^2†^

^*^ Equal contribution

^†^ Equal contribution

***Article type: Original research***

1. Department of Radiology, Kangbuk Samsung Hospital, Seoul, Republic of Korea
2. Department of Radiology, Seoul National University Hospital, Seoul, Republic of Korea
3. Department of Neurology, Seoul National University Hospital, Seoul, Republic of Korea

**Supplementary Materials**

**Figure S1.**

**
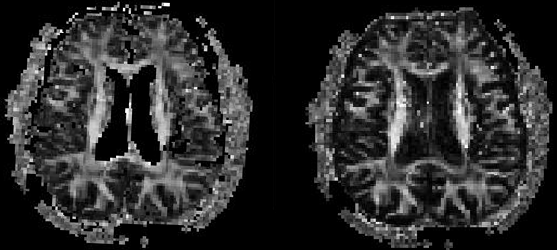
**

An example fractional anisotropy map acquired with (left) or without (right) free-water correction, in a same patient, same plane. Note the homogenous dark CSF space (indicating low fractional anisotropy) in the free-water corrected map.
